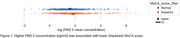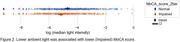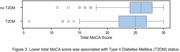# Environmental Factors in the Home Environment Associated with Lower Cognitive Function

**DOI:** 10.1002/alz70856_098636

**Published:** 2025-12-24

**Authors:** Jamie Shaffer, Aaron Y Lee, Cecilia S Lee

**Affiliations:** ^1^ University of Washington, Seattle, WA, USA; ^2^ The Roger and Angie Karalis Johnson Retina Center, Seattle, WA, USA; ^3^ Department of Ophthalmology, University of Washington, Seattle, WA, USA

## Abstract

**Background:**

Environmental factors and health have often been studied by geographical region; few studies have focused on indoor environmental quality. The Artificial Intelligence Ready and Equitable Atlas for Diabetes Insights (AI‐READI) project collects multimodal data from a diverse group of participants representing a range of Type 2 Diabetes Mellitus (T2DM) disease states (normal to insulin‐dependent). We used AI‐READI data to analyze associations between indoor environmental measures and early indicators of cognitive impairment.

**Method:**

Participants aged 40 and over with and without Type 2 diabetes are being recruited to the 4‐year AI‐READI project; data from the first 1067 individuals were released in year 2. Participants with Montreal Cognitive Assessment (MoCA) scores and environmental data were included. MoCA scores were binarized: 0 for scores > 25 (normal) and 1 for scores <=25 (impaired). Self‐reported T2DM status was binarized: 0 for no diabetes or lifestyle‐controlled diabetes, and 1 for insulin‐ or oral medication‐controlled diabetes. Environmental data were collected for 10 days in participants' homes using a device measuring volatile organic compounds (VOC), nitrogen oxides (NOX), relative light intensity, and particulate matter (PM).

**Result:**

Out of 1037 participants analyzed, 488 (47%) had impaired cognition. Higher PM concentrations (1, 2.5, 4, and 10 um or smaller) were associated with lower MoCA scores. For example, participants with cognitive impairment had a mean PM2.5 concentration of 21.53 [18.07, 24.99, 95% CI] compared to 11.53 [10.24, 12.82 95% CI] for normal participants. (Figure 1) Lower light levels were associated with lower MoCA scores: median light intensity was 0.03 [0.027, 0.040 95% CI] for normal participants and 0.02 [0.015, 0.026 95% CI] for those with impaired cognition. (Figure 2) We found significant associations between MoCA (binarized) and T2DM (Chi‐square, *p*‐value= 4.65e‐5). (Figure 3) There were no significant associations between cognitive function and VOC or NOX measures.

**Conclusion:**

Preliminary results from the AI‐READI cohort show an association between cognitive function (MoCA) and measures of ambient light, PM2.5, and T2DM status. Analysis of other factors (VOC, NOX) did not show significant associations.